# Braithwaite’s Retrospect

**Published:** 1862-01

**Authors:** 


Braithwaite's Retrospect.—Part XLIV, of this sterling publication is just received, too late for extract from its richly-stored pages. The same marked ability, discriminating taste and enlightened judgment characterize the present volume, as have won for its predecessors the favor and esteem of the profession. The *Commentary on Midwifery, and the Diseases of Women and Children*, by the senior Braithwaite, is one of the most valuable features, and a decided acquisition. The present number contains a general index for the volumes of 1860–1861. The publisher, (W. A. Townsend, 39 Walker Street, N. Y.), announces that the *Retrospect* has attained a permanent patronage, in this country, of five thousand regular subscribers, a prosperity it richly merits.

